# Electrophysiology of ionotropic GABA receptors

**DOI:** 10.1007/s00018-021-03846-2

**Published:** 2021-06-01

**Authors:** Erwan Sallard, Diane Letourneur, Pascal Legendre

**Affiliations:** 1 École Normale Supérieure de Paris, Biology Department, PSL Research University, 45 rue d’Ulm, 75005 Paris, France; 2 Department of Human Medicine, Faculty of Health, Center for Biomedical Education and Research (ZBAF), Institute for Virology and Microbiology, Witten/Herdecke University, 58453 Witten, Germany; 3 INSERM U1015, Gustave Roussy, Villejuif, France; 4 Master de Biologie, École Normale Supérieure de Lyon, Université Claude Bernard Lyon I, Université de Lyon, 69342 Lyon Cedex 07, France; 5 Sorbonne Université, UPMC Univ Paris 06, INSERM, CNRS, Neurosciences Paris Seine, Institut de Biologie Paris Seine (NPS, IBPS), 75005 Paris, France

**Keywords:** Synaptic receptor, GABA_A_ subtypes, Neurotransmitter, Neuronal inhibition, Phasic currents, Tonic activity

## Abstract

GABA_A_ receptors are ligand-gated chloride channels and ionotropic receptors of GABA, the main inhibitory neurotransmitter in vertebrates. In this review, we discuss the major and diverse roles GABA_A_ receptors play in the regulation of neuronal communication and the functioning of the brain. GABA_A_ receptors have complex electrophysiological properties that enable them to mediate different types of currents such as phasic and tonic inhibitory currents. Their activity is finely regulated by membrane voltage, phosphorylation and several ions. GABA_A_ receptors are pentameric and are assembled from a diverse set of subunits. They are subdivided into numerous subtypes, which differ widely in expression patterns, distribution and electrical activity. Substantial variations in macroscopic neural behavior can emerge from minor differences in structure and molecular activity between subtypes. Therefore, the diversity of GABA_A_ receptors widens the neuronal repertoire of responses to external signals and contributes to shaping the electrical activity of neurons and other cell types.

## Introduction

γ-Aminobutyric acid (GABA) is one of the main neurotransmitters in virtually all Metazoans. It is the most widely distributed inhibitory neurotransmitter in the central nervous system of mature vertebrates, being present in around 30% of the synapses [1, 2].

GABA-mediated signals can be transduced in receiving cells through metabotropic or ionotropic receptors located at the plasma membrane. GABA_B_ receptors are heterodimeric metabotropic receptors coupled to potassium and calcium channels through G proteins. On the other hand, GABA_A_ receptors are GABAergic pentameric chloride channels [3], i.e. ionotropic GABA receptors, belonging to the cys-loop family of ion channels [4]. Certain ionotropic GABA receptors were initially termed GABA_C_ receptors [5], but they are now classified as a subset of GABA_A_ receptors [6].

GABA_A_ receptors are found in all major taxa of bilateral metazoans [7] and are among the most abundant neurotransmitter receptors [8]. They are mainly found in neurons in synaptic, perisynaptic, and extrasynaptic locations. They can also be found in non-neuronal cells as well as outside of the nervous system.

The monomers constituting GABA_A_ pentameric receptors are drawn out of a large set of different subunits. Consequently, GABA_A_ receptors can be made of different combinations of subunits and are subdivided into subtypes: each subtype corresponds to a combination of subunits. Subtypes can differ in their electrophysiology and their pharmacology. The total number of functional subtypes existing in vivo may reach several hundreds [9], although most receptors belong to one or two dozens of major subtypes [10].

The conductance and current time-course of GABAergic receptor-channels depend on their subunits composition, the ionic conditions of their environment and the patterns of GABA exposure they face. In particular, the differential regulation, abundance and localization of GABA_A_ receptor subtypes enable a very precise modulation and a great diversity of responses to GABA signals across the brain and the organism, facilitating the numerous biological processes in which GABA_A_ receptors are involved.

Despite the complexity and biological significance of GABA_A_ receptors, recent reviews on their electrophysiology are missing. In this review, we summarize the current knowledge on the electrophysiology of the different GABA_A_ receptor subtypes and show how their properties explain the role they play in cellular and cerebral activity. We mainly focus on mammals and on neuronal GABA_A_ receptors. Related subjects such as GABA_A_ receptor pharmacology or pathology will not be covered in this review; other reviews have already been published about pharmacology [11–20], the regulation of subunits gene expression [21–23], subunit intracellular trafficking [24, 25], non-neuronal GABA_A_ receptors [26–29], and pathology [30–32].

## Diversity and structure of GABA_A_ receptors

### GABA_A_ receptor subunits diversity

Mammals have 19 genes coding for a GABA_A_ receptor subunit, classified in classes (α, β, γ, ρ, θ, ε, π and δ) and isoforms (α1–6, β1–3, γ1–3 and ρ1–3) [33, 34]. The diversity of subunits is further increased by alternative splicing, to which 9 out of 19 subunits are submitted [34, 35]. Alternative splicing enables the β2, γ2, γ3 and ρ1 subunit genes to produce at least two mature proteins, but differences in receptor activity depending on alternative splicing have been reported only between the γ2L and γ2S isoforms of the γ2 subunit; the main function of alternative splicing is believed to be the regulation of subunit expression [34].

Hundreds of millions of years ago, duplications of an ancestral gene led to the emergence of the different classes of subunits, and later to the different isoforms of a same class. The amino acid identity rate between isoforms of the same class ranges between 70 and 80%, whereas it typically lies between 30 and 40% for subunits of different classes [36].

Most subunits have remained very stable in the last tens of millions of years of vertebrate evolution. For example, the amino acid identity rate between mouse and human ortholog subunits is above 90% (except ρ3, with 84% identity). The conservation is not only structural but also functional: crustacean β subunits have been shown to functionally replace human β subunits in chimeric receptors [7, 37]. Likewise, deletion of a single GABA_A_ receptor subunit often results only in a mild pathology or no phenotype at all [38–42], showing that different subunits can substitute each other. These facts highlight the strong selective pressures applied on GABA_A_ receptors, and suggest it has a substantial biological importance.

### Subunits assemble into receptors whose structure is finely regulated

**Fig. 1:**
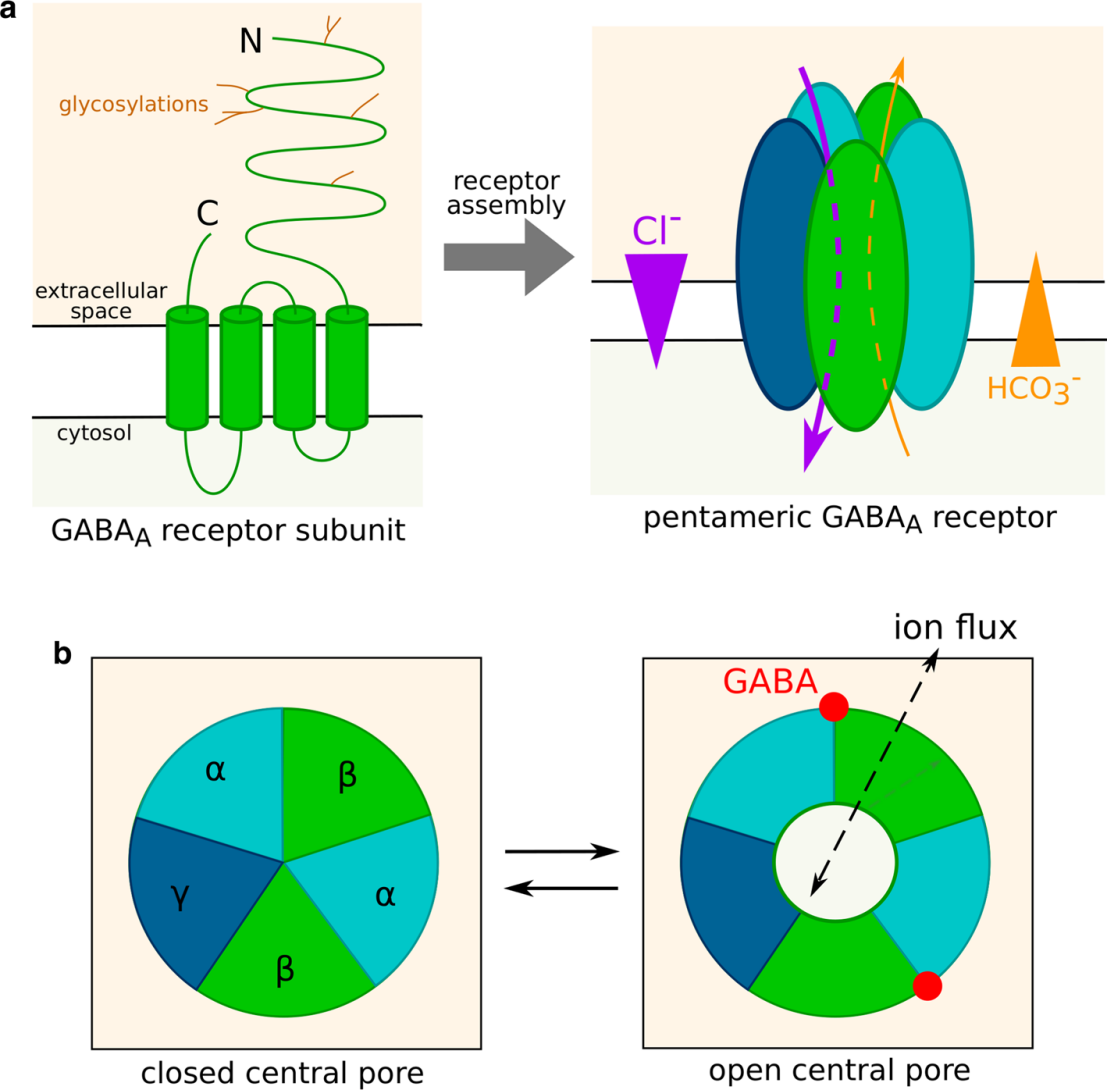
GABA_A_ receptor structure and gating. **a** Structure of an isolated GABA_A_ receptor subunit and of a mature receptor. Five subunits, potentially belonging to different classes or isoforms, assemble into a channel permeable to chloride and bicarbonate ions. Ions flow through GABA_A_ receptors down their electrochemical gradient. **b** Control of GABA_A_ receptor conductance by the receptor conformation. The central pore can be either closed or open as a result of agonist fixation on the receptor. In this example, the receptor is viewed from the extracellular space and the subunits are ordered as in the most abundant subtypes, namely αβγ. The presence of two GABA binding sites corresponds to ternary receptors (composed of subunits from three different classes)

Mature subunits are integral plasma membrane proteins. Human subunits are 420–632 amino acid long and weigh between 52 and 59 kDa [34, 36]. They contain an extracellular N-terminal segment of 220–250 residues, which can be glycosylated; 4 transmembrane domains termed M1, M2, M3 and M4; and a short extracellular C-terminal segment (Fig. 1a).

A functional receptor contains five subunits (Fig. 1a), assembled in a circle around a chloride-permeable pore delineated by the M2 transmembrane domain of all five subunits [8]. Each monomer is thus in contact with two other subunits, respectively at its “principle”, or + , and “complementary”, or −, faces.

The study of structure–function relationships of GABA_A_ receptors has long been hindered by the substantial diversity of conformations, making it difficult to stabilize a receptor population in a single state [43], and cryo-EM structures of GABA_A_ receptors were only recently resolved (reviewed in [44]). GABA-binding sites are located at interfaces between subunits on the surface of the extracellular part of the receptor. GABA binding induces a conformation change, typically when all binding sites are occupied, through a concerted rotation of the extracellular domains of the five monomers [45]. This rotation is translated to the transmembrane domains and results in variations of the opening of the central pore (Fig. 1b). Based on models of GABA_A_ receptor structure, Rossokhin predicted that the diameter of the channel pore is mainly controlled by residues—2′, 9′ and 20′ of the M2 transmembrane domain [46]. The ring formed by the 9′ M2 residues of the five subunits is termed activation gate. In the main closed conformation, the diameter of the ring is only 2.5–3.4 Å. GABA-induced conformation change prompts the side chains of the activation ring residues to shift away from the central pore, whose diameter increases up to 3.4–7.6 Å, allowing ions to flow through the channel.

The most frequent subunit composition of GABA_A_ receptors is 2 α, 2 β and 1 γ subunit [47, 48]. In this case, the 2 α and 2 β subunits in the receptor are most often, but not always, of the same isoform [48–53] and the receptor carries two GABA binding sites [54] located at the β+/α− subunit interfaces [44]. However, other stoichiometries can be observed [55]: ε, π and δ subunits can replace a γ subunit, and a θ subunit can replace a β subunit [33]. In addition, binary receptors (receptors containing two different classes of subunits instead of three, such as the αβ subtypes) are able to assemble in vitro. They were also described in vivo, but the evidence of their existence often comes from indirect methods [56] or study of γ2 or δ deficient mice [57, 58]: it is therefore possible that they are artefactual or accidental.

ρ subunits usually assemble in homopentamers or heteropentamers [59, 60], with five GABA binding sites per receptor [54], i.e. one at each subunit interface. However, in rare cases, ρ subunits assemble with α1 and/or γ2 subunits [61, 62].

### GABA_A_ receptors are classified into hundreds of subtypes

Due to the substantial subunit diversity, there are numerous subtypes of the GABA_A_ receptor, characterized by the combination of subunits they contain (e.g. α1β2γ3) [6].

GABA_A_ receptors are expressed in numerous cell types, most notably in neurons. The expression patterns of the different subunits determine which subtypes are assembled, as well as their cellular and subcellular location and their abundance. Numerous cells coexpress multiple GABA_A_ subunits isoforms [48–53]. Consequently, a single cell can express several GABA_A_ receptor subtypes [56, 63, 64].

The most abundant subtype in the mammalian nervous system is α1β2γ2, possibly accounting for 60% of all GABA_A_ receptors [65]. The other major subtypes are α2β3γ2 and α3β3γ2 [66], while α4–6βγ2, α6β2–3δ, α4β2–3δ and ρ1–3 are less abundant. Minor subtypes whose physiological existence in vivo is deemed likely include α1βδ, α2β1γ1, αβε, αβπ, αβθ, α1α6βγ2, α1α6βδ and αβγ3 [10, 66]. Though it is unlikely that all possible subunit combinations exist and assemble into functional receptors, the large number of possible combinations of subunits expressed by a given cell explains that the number of existing functional subtypes is estimated to be as high as 500 [9]. Most of these subtypes are expected be very rare compared to the most abundant ones [66] and to be limited to a specific tissue, brain area or developmental phase, but they may still exert non-neglectable effects on brain function because of the high abundance of GABA_A_ receptors compared to most neuronal receptors [9] and because rare subtypes can be selectively enriched in certain neurons or synapses [12].

## GABA_A_ receptor ion conductance selectivity

GABA_A_ receptors are ion channels. Different ions can flow down their electrochemical gradient through the pore from one side of the membrane to the other, generating currents. The intensity, i.e. the net charge transfer per time unit, of a transmembrane ion current is the product of the ionic electrochemical gradient by the number and conductance of open channels.

1 $$\frac{{{\text{d}}q}}{{{\text{d}}t}} = I = N_{{{\text{open}}}} \sum\limits_{{{\text{ions}}}} {\Delta E_{{{\text{ion}}}} G_{{{\text{ion}}}} } .$$

*N*_open_ is the number of open channels at the considered membrane; it depends on numerous parameters, notably GABA concentration. For each ion, Δ*E*_ion_ designates the cognate transmembrane electrochemical gradient and *G*_ion_ the conductance of a single open GABA_A_ channel.

The conductance of GABA_A_ receptors varies widely for different ions: we will study in this paragraph how this selectivity shapes the currents mediated by GABA_A_ receptors.

### GABA_A_ receptor chloride conductance

In most circumstances in the adult brain, K^+^ coupled secondary active Cl^−^ transporters [67] generate an outwardly directed chloride electrochemical gradient at the neuron plasma membrane [68]. Consequently, GABA-induced opening of GABA_A_ receptors induces a chloride influx (Fig. 1a), thus a hyperpolarization of the plasma membrane and an inhibition of action potential generation. This is the most frequent mechanism of inhibitory neurotransmission, which occurs in a large proportion of brain synapses.

However, during development [69], GABA_A_ receptors mediate chloride efflux due to an inverted electrochemical gradient [70], and thus often activate the neuron [70–73]. Such a mechanism has also been observed in slices prepared from adult brains [74], but these findings have been questioned because neurons in slices seem to have a higher intracellular chloride concentration than in vivo. This artifact may produce a non-physiological inversion of the chloride electrochemical gradient [75].

Membrane resistivity (*ρ*) is equal to the transmembrane potential (Δ*E*) divided by the intensity (*I*) of the current passing through a unit of membrane surface: *ρ = *Δ*E* × *S*/*I* (where *S* is the area of the membrane domain). The lower the resistivity is, the more intense is the transmembrane current generated by a given voltage gradient. The opening of channels, regardless if they mediate depolarizing or hyperpolarizing currents at the resting membrane potential, decreases membrane resistivity and promotes the dissipation of action potentials, a phenomenon termed shunting inhibition. Indeed, it enables ions to respond to the electrochemical gradient created by the action potential with local transmembrane flows instead of the expected longitudinal flows that are necessary for propagating the action potential to other membrane locations. Depolarizing currents mediated by GABA_A_ receptors can interfere with action potentials propagation through shunting [76] or inactivation of voltage-dependent Na^+^ channels [77], and can thus be inhibitory instead of excitatory [78, 79]. Conversely, hyperpolarizations mediated by GABA_A_ receptors may result in neuronal activation through rebound spikes [80, 81].

The GABA_A_ receptor-mediated depolarizations observed during development can induce an influx of calcium through voltage-dependent channels [80, 82], favoring neurite outgrowth [83], synaptogenesis [84], neuron migration [85], and neuron survival [86].

### Permeability of GABA_A_ receptors to ions other than chloride

Although chloride ions account for most of GABA_A_ receptor conductance in vivo [3], GABA_A_ receptors are also permeable to bicarbonate, formate, propionate, acetate, cyanide and halides [3, 4, 87]. Differences in GABA_A_ receptor conductance and permeability to these anions are caused by a selectivity filter relying on recognition sites [3] and the limited pore diameter.

Because of CO_2_ production and conversion in HCO_3_^−^ inside the cell, bicarbonate flows mediated by GABA_A_ receptors are outwardly directed (Fig. 1a), and thus depolarize the cell membrane. In standard physiological conditions, the ratio of HCO_3_^−^ vs Cl^−^ permeability of GABA_A_ receptors is comprised between 0.2 and 0.3 [88], i.e. chloride currents have a greater amplitude than bicarbonate currents and the net effect is hyperpolarization. However, prolonged GABA exposure can occasionally turn hyperpolarizing currents into depolarizing ones [89], notably during epileptic seizures or in hypothalamic hamartoma neurons [90]. This phenomenon probably indicates a dissipation of the chloride gradient [91, 92] when the channel remains open for long periods. Under these conditions, the bicarbonate efflux may become larger than the chloride influx and GABA_A_ receptors may transiently become excitatory [72].

## Intrinsic electrophysiological parameters of the different GABA_A_ receptor subtypes

**Fig. 2:**
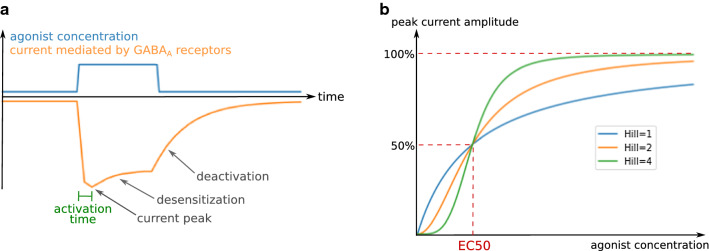
General characteristics of GABA_A_ receptor-mediated currents. **a** Typical GABA_A_ receptor current time-course and kinetic electrophysiological parameters. **b** Dependence of GABA_A_ receptor-mediated current intensity on agonist concentration for different Hill coefficients

The impact of GABA_A_ receptors on cellular electrophysiology mostly depends on the time-course and amplitude of the chloride currents evoked by exposure to an agonist such as GABA. Typically, the current time-course following the introduction of an agonist in the receptor’s environment consists of an initial current peak, followed by a decrease in amplitude (Fig. 2a). This decrease ends with a stabilization at lower amplitude or a complete closure of the receptors, depending on agonist concentration, duration of exposure to the agonist, and on the receptor subtype. The effects of GABA_A_ receptors on neuronal activity are determined primarily by the charge transfer (the product of the current intensity with its duration) [93], but also by its synchronization with other electrical inputs, hence the importance of the shape of the current time-course [94, 95]. The current intensity at a certain time point is the product of the conductance of a single channel by the chloride electrochemical gradient and the number of open receptors (Eq. 1), which depends on kinetic parameters governing channel gating as well as receptor and agonist concentrations. Indeed, the effects of an agonist are dose-dependent: for example, the proportion of open receptors as a function of the GABA concentration follows an allosteric pattern (Fig. 2b), characterized by a concentration threshold at which the proportion of open receptors sharply increases.

In most physiological situations, the cell response to a GABA input is driven by the joint action of dozens or hundreds or receptors that perceive the signal. The macroscopic scale (receptor populations) therefore appears more suited than the microscopic scale (single channels) to understand the biological consequences of GABA_A_ receptor electrophysiology and is easier to study, which explains that most available information on GABA_A_ receptor electrophysiology covers macroscopic features. Nevertheless, macroscopic properties always emerge from the integration of single channel activity characteristics.

The electric activity of GABA_A_ receptors can be understood with a series of parameters that describe and explain their responses to the various patterns of agonist exposure. In this section, we will describe the macroscopic electrophysiological parameters that determine the activity of populations of GABA_A_ receptors in response to GABA, and we will highlight the differences between subtypes in that regard.

Electrophysiological parameters can be subdivided into kinetic parameters (activation time, desensitization time, deactivation time…) and so-called functional parameters (conductance, EC50, Hill coefficient…), which describe respectively the current time-course and its dependence on agonist concentration. As will be exemplified below, some of these parameters are not independent.

### Tools for the study of GABA_A_ receptor electrophysiology

Before detailing the electrophysiological parameters of GABA_A_ receptors, we will discuss two conceptual tools that were instrumental in the discovery and understanding of these parameters: modeling and the use of channel modulating drugs.

#### Modeling

Numerous models have been used to interpret the growing amount of data on GABA_A_ receptor electrophysiology, predict receptor activity, and understand the microscopic origin of macroscopic parameters and large-scale behavior of channel populations.

**Fig. 3:**
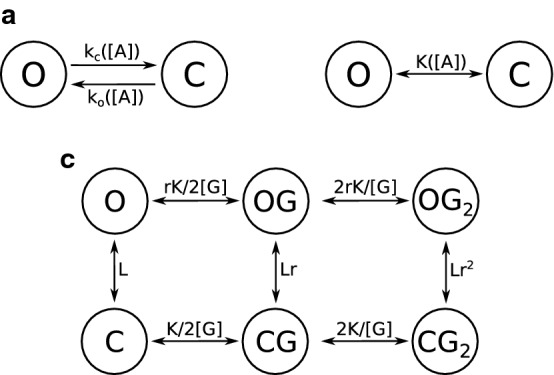
Markovian models of receptor channel activity. **a** Simplest Markovian model of a receptor channel, with two unitary states: open (O) and closed (C). The transition rates describing channel opening and closure are respectively *k*_o_ and *k*_c_. Both transition rates are a function of the agonist concentration [*A*]. **b** Application of the two-states model to steady-state situations. *K* is the equilibrium constant, and depends on agonist concentration. **c** MWC model. *L*, *r* and *K* are constants depending on receptor structure and [G] is the GABA concentration. The open states are O (no GABA bound), OG (one GABA) and OG_2_ (two GABA); likewise, C, CG and CG2 are the close states

Most of the models used are Markovian. Markovian models are discrete (time is discontinuous) and memory-free: the next step of evolution of a model depends only on its present state, and not on past states. Markovian models describe the activity of a channel with a series of states, each of them having its own conductance and being supposed to correspond to a specific receptor conformation. Transition rates can be a function of environmental parameters such as transmembrane potential or agonist concentrations. Transition rates indicate the kinetics of channel activity as well as states occupancy at equilibrium. For example, in the model showed in Fig. 3a, if *p* is the proportion of receptors in the open state at a given time point, a proportion *pk*_c_ of the receptor population transitions to the closed state before the next time point, while a proportion (1 − *p*)*k*_o_ of receptor transitions to the open state. At equilibrium, the net flux is null, meaning that *pk*_c_* = *(1 − *p*)*k*_o_, and therefore *p = k*_o_/(*k*_c_* + k*_o_).

Certain models specifically describe systems at equilibrium. This simplification allows the replacement of transition rates by equilibrium constants. In our example model, the equilibrium constant K would follow the equation *K = p*_eq_/(1 − *p*_eq_) = *k*_o_/*k*_c_ (Fig. 3b).

The most classic model used to describe GABA_A_ receptor activity is the Monod–Wyman–Changeux two-state concerted transition model (MWC) [96]. This equilibrium model takes into account one open and one closed conformations, both of which can be bound to zero, one or two GABA molecules (Fig. 3c). The MWC model requires only four parameters to entirely describe the receptor response to an agonist, which explains its attractiveness. It accurately describes GABA_A_ activity at the macroscopic scale, and its activation by numerous agonists and combination of agonists [47, 96–98]. This model has confirmed the presence of two GABA binding sites on ternary receptors [97].

Other models (Table 1) complexify the MWC model to fit more accurately to certain experimental data, such as the flip model, which includes a transient pre-sensitized closed conformation with high agonist affinity [96, 99]. More complex models [100–104] consider a greater number of states. Although generally more accurate than simpler models, complex models often lack predictive value since it is difficult to accurately estimate all the parameters of the model [96].

**Table 1:** Examples of Markovian steady-state models used for GABA_A_ receptor study

Model	States	Conditions of application
MWC	Close, open (both bound to 0, 1 or 2 GABA)	Macroscopic scale
Flip model	Resting, resting bound to GABA, pre-sensitized bound, open bound	Macroscopic scale
Resting-activated-desensitized	Resting, open, desensitized (all bound to 0, 1 or 2 GABA)	Desensitizing receptors
Chang and Weiss model	Close (bound to 0–3 GABA), open	ρ-Containing receptors

Complex models usually stem from the study of single channels, whose complex behavior requires several states to be accurately described, whereas fewer states are necessary to describe the properties of GABA_A_ receptors activity at the synaptic or cellular scale. Despite the likely existence of several conformations, the two-conformations MWC models provide accurate predictions at a macroscopic scale. This indicates that the receptor conformations can be separated into two groups with minor electrophysiological differences within each group [96, 105], or that some of them are occupied only transiently and are not involved in the steady-state circumstances that the MWC model is used to describe.

Electrophysiological parameters can be calculated as a function of transition rates and state occupancies in certain models. Modeling also helps to understand the interdependence between parameters, or to calculate parameters inaccessible to direct experimentation. For example, Chang and Weiss [106] estimated that ρ-containing receptors open when they bind GABA in at least 3 of their 5 binding sites.

#### Channel modulating drugs

The activity of GABA_A_ receptors is modulated by subtype-selective agonists and antagonists belonging to different chemical families such as benzodiazepines or barbiturates and including anesthetic, anxiolytic or antiepileptic drugs.

Since primary and transfected cells can often express several subtypes simultaneously, it is necessary to distinguish from which subtype come the currents measured during electrical recordings. Subtype-selective channel modulating drugs facilitate in situ subtype identification [10].

For example, Lindquist and Birnir performed a patch-clamp single-channel study of GABA_A_ extrasynaptic receptors on dentate gyrus granule neurones and identified three types of electrophysiological signatures [107]. Receptors of the first, second and third type were potentiated respectively by zolpidem, flumazenil and THDOC, indicating that they belong respectively to α1βγ2, α4βγ2 and αβδ subtypes.

Channel modulating drugs have also been used to study GABA_A_ receptors structure and to map subtypes expression in the brain. The benzodiazepine molecules [3H]Ro15-4513 and diazepam bind respectively all γ-containing subtypes and α1,2,3,5βγ receptors. Hence, under competition from diazepam, [3H]Ro15-4513 binds only α4,6βγ subtypes. Using this property, Korpi et al*.* mapped α4,6βγ receptors by autoradiography of [3H]Ro15-4513 in mouse brains and showed that they were overexpressed in δ−/− mice [108]. This proved that γ subunits can partly substitute δ.

Etomidate is a general anesthetic binding GABA_A_ receptors on their GABA binding sites and potentiating their response to GABA. This potentiation involves comparable allosteric shifts between αβδ and αβγ subtypes, showing that αβδ subtypes carry two GABA binding sites as had been previously described for αβγ subtypes [109].

### Kinetic parameters

#### Activation time

The delay between a change in GABA concentration and channel response varies between receptor subtypes [110]. The channel opening occurs in two phases: first, agonists bind to the receptor; second, the receptor conformation changes and the central pore opens. Thus, the delay in GABA_A_ receptor opening upon GABA introduction in its environment depends both on GABA binding kinetics [111] and on the kinetics of receptor conformational change upon GABA fixation [111]. GABA fixation kinetics is determined by GABA concentration [75, 100, 105], following an allosteric pattern [112], and by the receptor affinity for GABA. At a given GABA concentration, the characteristic time of the GABA binding kinetics is theoretically inversely proportional to the receptor affinity for GABA: this relation has been observed in β2γ2S-containing subtypes [75]. However, at saturating GABA concentrations (in the order of 10 mM [112]), the conformational change kinetics becomes the limiting factor. This may explain why the δ subunit lowers the reactivity of the receptor compared to γ2 [113] despite a higher affinity. The activation time, i.e. the time to go from 10 to 90% of open channels after introduction of GABA, is 0.46 ms for α1β3γ2L at a GABA concentration of 1 mM, versus 2.4 ms for α1β3δ [114].

The activation times provided in Tables 2, 3 and 4 are calculated at saturating GABA concentration, thus should reflect the kinetics of conformation transition. In association with β3 and γ2L subunits, the rank order of α subunits in terms of activation time is α1 < α2 < α4 < α6 < α5 < α3, with α3 being three times slower than α1 [110–112, 114]. However, when associated with β1 and γ2, α2 confers an activation twice faster than α1 [100]. αβ subtypes [115], and even more ρ-containing subtypes [116], display an extremely slow activation time lasting several tens of milliseconds. Receptors containing the ε subunit display slow activation in vivo [117]. However, this property has never been studied with recombinant ε-containing receptors, thus it may be conferred by another subunit.

The measured activation time depends on the size of the experimental settings. For example, macro-patches, in which several receptors gathered on a micrometric membrane domain are studied, display faster activation than whole-cell recordings [112]. It may be due to the time required for GABA to diffuse to all GABA_A_ receptors, which is longer for larger experimental models, or to cytoplasmic proteins that may modulate GABA_A_ receptor kinetics in whole-cell studies [112].

#### Desensitization

**Fig. 4:**
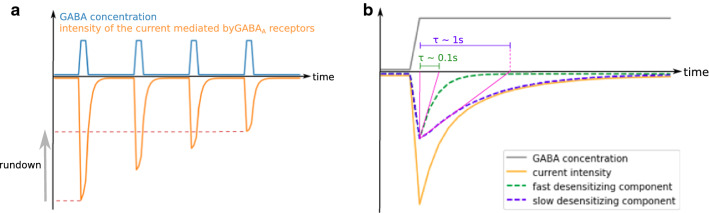
Characteristics of GABA_A_ receptor desensitization. **a** Rundown. When GABA_A_ receptors are exposed to frequent GABA transients, a portion of the receptor population desensitizes at each transient and does not recover before the next exposure, resulting in a decrease in peak amplitude. **b** Biphasic desensitization. The current decay upon desensitization can often be modeled as the sum of two decreasing exponential components. A global desensitization rate can be computed from the characteristic time (*τ*) of each component.

Upon long exposure to GABA, GABA_A_ receptors lose their ability to open: this process is called desensitization (Fig. 2a). This mechanism is also observed when receptors are exposed to GABA transients at a high frequency and is then termed rundown (Fig. 4a) [118]. Desensitization prevents the transmission of abnormally strong inhibitory signals during synaptic communication: if GABA is chronically present in the synapse rather than by interspaced bursts, as it is under normal conditions, postsynaptic GABA_A_ receptors will close [119].

In desensitized conformations, the M2 transmembrane domains are tilted and their -2′ residues obstruct the intracellular end of the central pore of GABA_A_ channels [46]. The five monomers constituting the channel can transition between open and desensitized conformations independently from one another [120].

During prolonged GABA exposure, the proportion of open channels sometimes follows a decreasing exponential curve over time [75], thereby a characteristic time of desensitization (*t*_d_) can be calculated. In other cases, the observed desensitization curve can be described as the addition of several exponential components [112, 121, 122] (Fig. 4b). In this situation, a single *t*_d_ can be computed as the average of the characteristic times of each component weighted on the amplitude of the related current (this is the procedure followed to calculate the *t*_d_ shown in Tables 2, 3 and 4). Consequently, the calculated value of *t*_d_ depends on the duration of GABA exposure [111]. In long exposures, slow components gain in relative importance and the calculated *t*_d_ increases. In addition, *t*_d_ depends strongly on GABA concentration [75]: the curve of desensitization rate as a function of GABA concentration follows approximately an allosteric pattern. When enough data are available for calculations, the *t*_d_ presented in Tables 2, 3 and 4 are the asymptote of this curve at infinite GABA concentration.

The characteristic time of desensitization is usually in the order of 1 s, despite important variations. αβγ receptors containing the α4 subunit desensitize two to three times faster than those containing the α3 or α5 subunit, with α1 and α2 desensitizing almost as fast as α4, and α6 conferring an intermediary desensitization time [75, 111, 112]. However, when the receptor includes a δ subunit, α6 appears to confer faster desensitization than α1 [123]. β2 confers a slightly faster desensitization than β3, with β1 as an intermediate [124] or giving results similar to β2 [100]. Desensitization of γ2-containing receptors is two or three times faster than that of δ-containing receptors [113, 123, 125], but substantially slower than that of ε-containing receptors [126].

Certain subtypes do not desensitize or desensitize only partially. For example, homomeric ρ-containing receptors are almost insensitive to desensitization [5, 116, 127, 128]. Unlike γ-containing subtypes, receptors containing the δ subunit desensitize only partially [101]: α1β3δ currents decrease by only 11.6% upon 6 s exposure to saturating GABA concentration [123]. The α subunit plays a lesser but still notable role in determining the extent of desensitization (Table 5): γ-containing subtypes desensitize incompletely only if they contain the α3 or α5 subunit [111], with a steady-state current in prolonged GABA exposure of up to 30% of the peak amplitude. α1β3 receptors containing two α and three β subunits desensitize more extensively than receptors containing three α and two β subunits [129].

Desensitization can be followed by a refractory period [114], during which receptors are unable to reopen. The length of this period is modeled as a function of several decreasing exponentials, indicating that GABA_A_ receptors can adopt several closed conformations [114, 130], whose relative proportions depend on GABA concentration [130]. In vivo, the fast components of the refractory period disappear at low GABA concentration. In these conditions, desensitization is less extensive but its effects last longer [130, 131]. The length of the refractory period varies between subtypes, with receptors containing α4 or α5 being the fastest to recover (with a characteristic time of 25 ms following a 5 ms exposure to GABA), followed by α2 and α3 (about 100 ms), then α1 (about 200 ms) and finally α6 (about 400 ms) [111]. Nonetheless, characteristic times heavily depend on experimental procedures and method of calculation [112].

The study of desensitization prompted the development of complex, non-steady-state models of GABA_A_ receptor activity since the MWC model does not describe receptor desensitization and therefore applies well only to non-desensitizing subtypes or to short time-frames of activation. These limitations are tackled by three-state models such as the resting-activated-desensitized model (Table 1) [132]. Other models describe the different components of desensitization or refractory periods with several desensitized states [114, 120, 133]; the desensitization times and durations of refractory period components are represented by the transition rates respectively to and from these states. Using one of these models, Gielen et al*.* [120] proposed that the fast component of desensitization corresponds to receptors with two of their five monomers adopting a desensitized conformation, while the slow component corresponds to receptors with at least three desensitized monomers.

Speed, extent and relative proportions of exponential components of desensitization are critical in shaping the time-course of the inhibitory post-synaptic current (IPSC) mediated by GABA_A_ receptors and strongly differ between subtypes [102, 106, 114, 134, 135]. For example, α6β3δ has no fast component, unlike α6β3γ2L [101, 123]. Consequently, despite the similarity of the characteristic time of their slow component of desensitization, α6β3δ appears to desensitize less extensively than α6β3γ2L and is less sensitive to the duration of GABA exposure. As a result, α6β3δ transmits inhibitory signals whose intensity is roughly proportional to the duration of GABA exposure, while α6β3γ2L-mediated signals do not depend on exposure duration. This exemplifies that minor differences between subtypes at the microscopic scale can be amplified at larger scales. Furthermore, the time-course of IPSCs determines the strength and timing of neuronal inhibition, so limited differences in desensitization properties can have dramatic effects in the response of neuronal circuits to high-frequency inhibitory signals [101, 136].

#### Deactivation

GABA_A_ receptors spontaneously release their ligands and close several milliseconds or tens of milliseconds after a drop in GABA concentration. The event of deactivation puts an end to the transmission of a hyperpolarizing signal during synaptic communication, after a brief GABA exposure (synaptic GABA bursts usually last no longer than a few milliseconds). Contrary to desensitized channels, deactivated channels can reopen without a refractory period.

Deactivation has been observed to be biphasic in vitro [100, 103, 111, 114, 122, 133] and in vivo [105, 137]; the relative proportions of the two components can vary depending on the receptor subtype. These two components may correspond to two conformations, one bound to two GABA molecules (the slow component) and the other to a single one (the fast component) [102], but this hypothesis is debated [114]. Deactivation can sometimes be triphasic [56], once again depending on the subtype and probably on recording procedures, because certain protocols do not enable the recording of very fast components [112].

Homomeric ρ-containing receptors deactivate quickly [116]. Among α-containing subtypes, those containing α4 are the fastest to close, followed by α5 (1.5 times slower), α1 (2.5 times slower than α4), then α2 (5 times slower than α4), and finally α6 (6 times slower than α4) and α3 (about 10 times slower than α4). The γ2 subunit confers a slower deactivation than δ [138, 139]. The β2 subunit confers a substantially faster deactivation than β3 [140].

**Fig. 5:**
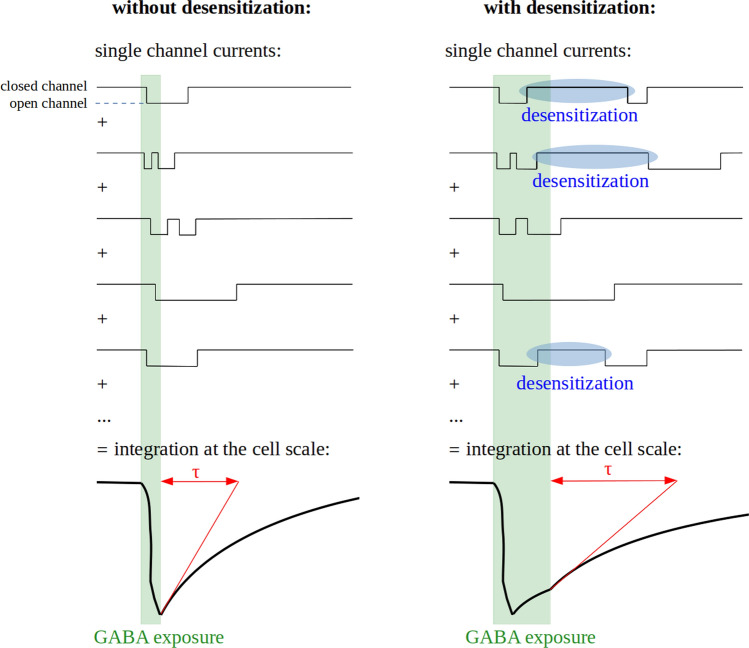
Relationships between microscopic and macroscopic kinetics of channel opening. Deactivation is a macroscopic property resulting from the integration of single-channel currents time-courses. Its characteristic time (*τ*) is longer than mean channel open time due to possible channel reopening events and is increased by desensitization due to the possible reactivation of desensitized channels

Most studies on deactivation have been conducted on whole-cell or multichannel patches, which should give results close to what is observed in vivo at the synaptic or cell scale. However, deactivation is much faster in single-channel (0.2–25 ms) than in channel cluster or whole cell (5–680 ms) recordings [141, 142]. This discrepancy likely stems from sequential closing and opening events of a single channel (Fig. 5), which are integrated in a single current time-course by whole-cell or multichannel patches recordings [105, 143]. It exemplifies the apparent gap between the complex macroscopic properties of GABA_A_ receptors and the single-channel activity they emerge from.

The deactivation time increases in proportion with the extent of desensitization [102, 123, 135], maybe due to a high GABA affinity of desensitized states [135]. GABA is thus retained on desensitized or pre-desensitized receptors for tens of milliseconds, even when all GABA has been cleared from the extracellular space [144]. If the receptor is still bound to GABA when the refractory period ends, it is able to reopen immediately. When numerous receptors are considered together, this process results in a prolongation of the phase in which the current decreases, which is described as a slower deactivation (Fig. 5). Consequently, the fast components of desensitization prolong GABA_A_ receptor responses to GABA and therefore synaptic communication while decreasing the current amplitude [102, 131].

### Functional parameters

#### Conductance

A single GABA_A_ channel can display several conductance states, remarkably conserved between species and cell types, ranging around respectively 12, 20, 30 and 45 pS for the most frequent subtypes. The main conductance state is 30 pS, meaning that this is the state adopted by the channel in most circumstances when it is open [4]. The conductance states may correspond to different channel conformations.

Most GABA_A_ receptor subtypes display very similar conductance values (Tables 2, 3, 4) [145, 146]. However, the main conductance state of γ-containing receptors (~ 30 pS) [125] is higher than that of δ-containing receptors (~ 22 pS) [125], ρ-containing receptors (7 or 8 pS) [147, 148] and αβ binary subtypes (11–20 pS) [56].

#### GABA EC50

The curve of GABA_A_ receptor peak current intensity as a function of GABA concentration follows an allosteric pattern (Fig. 2b), described by the equation of Hill:

2 $$\frac{I}{{I_{{{\text{max}}}} }} = \frac{{[A]^{h} }}{{{\text{EC}}50^{h} + [A]^{h} }},$$

where *I* is the current intensity, *I*_max_ its asymptote at infinite agonist concentration, [*A*] the agonist concentration, and *h* the Hill coefficient (see “Hill coefficient”).

EC50 is the GABA concentration at which the current peak reaches 50% of its maximal amplitude. The EC50 partially reflects the receptor affinity for GABA: in general, the lower the EC50, the higher the affinity. However, the EC50 also depends on gating parameters such as the speed or maximal probability of channel opening. This can be exemplified by a simple stochastic model with a single binding site and a single opening state.

3 $$A + R\begin{array}{*{20}c} {K_{{{\text{on}}}} } \\ \rightleftharpoons \\ {K_{{{\text{off}}}} } \\ \end{array} AR\begin{array}{*{20}c} \beta \\ \rightleftharpoons \\ \alpha \\ \end{array} AR*,$$

where *A* is an agonist that binds to a receptor site *R*, *A* + *R* is the vacant state of the receptor site, *AR* the occupied state, AR* the open state, *K*_on_ and *K*_off_, respectively the association and dissociation constants of the agonist, and *β* and *α* are respectively the opening rate and closing rate constants of the channel. In this model, the true affinity of the receptor for its ligand is the microscopic dissociation constant *K*_d_ = *K*off/*K*_on_. The receptor efficacy is defined as the equilibrium constant of the closed-open isomerization *E* = *β*/*α*. In this model, the apparent affinity EC50 verifies EC50 = *K*_d_/(1 + *E*).

The receptor efficacy is related with Pomax, the maximum fraction of receptors in the active state, by the relation Pomax = *E*/(1 + *E*) = 1 − (EC50/*K*_d_). Pomax can only be estimated at saturating GABA concentration using non-stationary noise analysis [149]. However, the presence of several open states can render the estimation of receptor efficacy very challenging. Hence, the true affinity and efficacy of GABA_A_ receptors are often inaccessible to accurate measurements and the EC50 is used as a measure of the apparent affinity.

When certain receptors of a cluster open for the first time upon GABA exposure, others may be already closed, due to fast desensitization. It results in a smoothened shape of the current time-course through decreased peak amplitude and spreading of charge transfer over time. This phenomenon is stronger at high GABA concentrations, which explains why Pomax is often substantially inferior to 1 and EC50 is close to the true affinity *K*_d_.

GABA EC50 is up to 100-fold lower than the concentration (termed BC50) at which the probability of occupancy of the GABA binding sites is equal to 0.5 [150], and than the concentration at which the half-maximal activation rate is reached [100, 112]. This discrepancy is explained by differences in GABA affinity between receptor conformations [106] and by the influence of desensitization on EC50, as confirmed by the small difference between EC50 and BC50 in the non-desensitizing ρ1 subtype [106]. It indicates that the EC50 is not a purely thermodynamic parameter but partially depends on receptor kinetics. Macroscopic parameters of GABA_A_ receptor electrophysiology are complex and emerge from the interconnection of the numerous properties of single channels.

Significant differences in GABA EC50 can be observed between GABA_A_ receptor subtypes, covering 2 orders of magnitude from around 0.5 µM to around 50 µM. Discrepancies are also observed for the same subtype in different studies, due to differences in the cell type where the receptor was expressed, in the species of origin of the receptor subunits or in experimental design (Tables 2, 3, 4). It nonetheless appears that the α subunit plays a crucial role in determining the receptor’s affinity for GABA (Table 5). The rank order of increasing GABA EC50 is α6 < α1 ~ α2 < α4 < α5 < α3 when these subunits are associated with β3 and γ2S [151], but α6 < α4 ~ α5 < α1 < α2 < α3 in association with β3 and γ2L [111]. The β isoform affects GABA affinity to a limited degree, with the β3 isoform conferring a one to fourfold lower GABA EC50 than β1 and β2 (Table 6). The γ1 subunit confers a higher EC50 than γ2 when these subunits are associated with α5, but in association with α3, the relationship is inverted. Several studies indicate a low EC50 in receptors containing the γ2S or γ3 subunits compared with the γ2L splice variant (Table 7). Receptors containing the δ subunit consistently display an EC50 around 5 times lower than γ2-containing receptors. ρ-containing subtypes are around 7 times more sensitive to GABA than the most abundant ternary receptors [147]. Subtypes containing two different isoforms of the same class generally show intermediate properties between the two corresponding subtypes containing only one of these two isoforms [125, 152–157]. Binary receptors have a very low GABA EC50 [146, 158], around 1 µM, which is as low as the most GABA sensitive ternary receptors. Binary α1β3 receptors containing two α and three β subunits have a lower GABA EC50 than α1β3 receptors with three α and two β subunits [129].

#### Hill coefficient

The Hill coefficient of a GABA_A_ receptor depends on its number of GABA binding sites and on cooperativity between GABA binding sites, the process by which the binding of GABA to one site increases the affinity or accessibility of the other site(s) for GABA. The higher the number of GABA binding sites or the extent of cooperativity are, the higher the Hill coefficient is. For Hill coefficients higher than 1, there is a threshold in GABA concentration where the opening probability of the receptor dramatically increases over a limited concentration span. The higher the Hill coefficient, the steeper the threshold (Fig. 2b).

In experimental systems with low temporal resolution, fast desensitization can decrease the resolution of the current peak, which might lead to errors in calculations of Hill coefficients. To the best of our knowledge, this phenomenon has not been tested with GABA_A_ receptors, but it was described in the glycine receptor [159] which belongs to the same receptor superfamily as GABA_A_ receptors.

Most GABA_A_ receptor subtypes have very similar Hill coefficients of around 1.5. However, binary αβ receptors and ε-containing receptors display low cooperativity [126, 146, 160, 161] with Hill coefficients below 1. On the opposite, ρ-containing subtypes display higher Hill coefficients than the most abundant ternary receptors [106, 128, 162], probably due to the presence of 5 GABA-binding sites instead of 2 [54].

### Subunits interplay in the integration of subtype-specific electrophysiologycal properties

 Due to the remarkable evolutionary conservation of the sequence of GABA_A_ receptor ortholog subunits, the electrophysiological properties of a certain subtype are expected to be similar in different species. To our knowledge, receptors of the same subtype but different species have never been compared in the same study. Tables 2, 3 and 4 highlight significant differences in the main parameters of certain subtypes between species, but important intraspecies differences are also observed, sometimes even when the subunits are expressed in the same cell line. Indeed, experimental procedures and measurement techniques can have a substantial influence on the results [112]: it can therefore be difficult to compare different subtypes. A further complication is that many authors did not distinguish between the splice variants γ2S and γ2L. Still, conclusions can be drawn by comparing subtypes studied in the same article with the same protocols or when several articles give very similar results on the same subtype.

**Table 2:** Main electrophysiological parameters of the different GABA_A_ receptor subtypes in standard ionic conditions (subunits expressed in *Xenopus* oocytes)

GABA_A_ receptor subtype	Single-channel conductance (pS)	GABA EC50 (µM)	Hill coefficient	Characteristic time of desensitization *t*_d_ (s)	Characteristic time of deactivation (ms)	Activation time (ms)
α1β1γ1		1.2 [167]				
α1β1γ2	**30 **[100]	4.5 [167] **6 **[100]	**1.2 **[100]	**0.492 **[100]** ([GABA] = 1 mM)** **0.6 **[103]** ([GABA] = 100 µM)**	**20.5 **[100] **17.4 **[103]	**1.1 **[100] **1 **[103]** (γ2S splice variant)**
α1β1γ3		0.6 [167]				
α1β2γ1		0.67 [167] (human γ1, rat α1 and β2, expressed in 293 cells)				
α1β2γ2	32 [152] ***27.9 ***[56]*** (γ2S splice variant)***	5 [172] 7 [110] (γ2S splice variant) **1.71 **[177]	1.6 [110] (γ2S splice variant) 2.12 [152]	1.4 [110] (γ2S splice variant)	220 [110] (γ2S splice variant)	
α1β2γ3	30.2 [178]	1.3 [167]				
α1β2δ		**6.71 **[179]				
α1β3γ1		2.1 [167]				
α1β3γ2	***28 ***[56]*** (γ2S splice variant)*** **27.5 **[180]	1.7 [167] 2.9 [151] (γ2S splice variant) 7.6 [181] (γ2L splice variant) 14 [112] (γ2L splice variant) **36.2 **[158]** (γ2L splice variant)** **14.1 **[122]** (γ2L splice variant)**	**1.7 **[158]** (γ2L splice variant)**	0.424 [111] (average of 1 and 3 mM of GABA, γ2L splice variant) 0.95 [112] (γ2L splice variant, 1 mM of GABA)	68.6 [111] (γ2L splice variant) 143 [112] (γ2L splice variant) **82.5 **[122]** (γ2L splice variant)**	0.55 [101] (γ2L splice variant) 0.603 [111] (γ2L splice variant)
α1β3δ	**27.5 **[180]	7.4 [181]			63.4 [181] 86.8 [123]	
α2β1γ2	**30 **[100]	**17 **[184] **37 **[100]	**1.8 **[100]	**0.449 **[100]** ([GABA] = 1 mM)**	**198.7 **[100] **82 (type 1) or 51 (type 2) **[103]	**0.5 **[100] **0.5 **[103]** (γ2S splice variant)**
α2β1ε		**11.2 **[185]				
α2β2γ2		**1.47 **[177]				
α2β3γ2		5.2 [151] (γ2S splice variant)		**0.391 **[111]** (average of 1 and 3 mM of GABA, γ2L splice variant)**	**110.6 **[111]** (γ2L splice variant)**	**0.735 **[111]** (γ2L splice variant)**
α3β1γ2		15.1 [167] (human α3, rat γ2 and β1, expressed in 293 cells)				
α3β2γ2		130 [187] 15.1 [167] (human α3, rat γ2 and β2, expressed in 293 cells) 75 [110] (γ2S splice variant) **2.21 **[177]	1.5 [110] (γ2S splice variant) 1.1 [187]	3.2 [110] (γ2S splice variant)	680 [110] (γ2S splice variant)	
α3β3γ2		48 [151] (γ2S splice variant)		0.701 [111] (average of 1 and 3 mM of GABA, γ2S splice variant)	188.8 [111] (γ2L splice variant)	1.788 [111] (γ2L splice variant)
α4β1γ2		**1.40 **[177]				
α4β1δ		**0.30 **[188] **0.17 **[177]	**1.57 **[188]			**174 **[177]
α4β2γ2		3.9 [189] 1.4 [167]				
α4β2δ		**0.25 **[179]				
α4β3γ2		7.6 [151] (γ2S splice variant) 15 [112] (γ2L splice variant)		0.408 [111] (average of 1 and 3 mM of GABA, γ2L splice variant) 0.711 [112] (γ2L splice variant, 1 mM of GABA)	24.8 [111] (γ2L splice variant) 109 [112] (γ2L splice variant)	0.951 [111] (γ2L splice variant)
α4β3δ		**0.35 **[188]	**1.8 **[188]			
α5β1γ2		5.6 [167]				
α5β2γ2		5.8 [187] 4.2 [167] **0.47 **[177]	1.5 [187]			
α5β2γ3		4.9 [190]	1.9 [190]			
α5β3γ2	**24.9 **[180]	11.6 [151] (γ2S splice variant)		1.315 [111] (average of 1 and 3 mM of GABA, γ2L splice variant)	41.5 [111] (γ2L splice variant)	1.247 [111] (γ2L splice variant)
α6β1γ2		0.5 [167]				
α6β2γ1		0.4 [167]				
α6β2γ2		5.2 [156] (γ2S splice variant) 1.4 [189] 0.34 [172] **0.50 **[179]** (γ2S splice variant)**				
α6β2δ		0.52 [156] (γ2S splice variant) 1.2 [167] **0.21 **[179]				
α6β3γ2		1 [151] (γ2S splice variant)		0.596 [111] (average of 1 and 3 mM of GABA, γ2L splice variant)	163.8 [111] (γ2L splice variant)	1.052 [111] (γ2L splice variant)
α6β3δ					340.4 [123]	
ρ1		**1.7 **[116]	**3.5 **[116]	**14.5 **[116]** (30 µM of GABA)**		**154 **[116]
ρ2		**2.6 **[116]	**3.3 **[116]	**8.2 **[116]** (30 µM of GABA)**		**180 **[116]

Roman: rat subunits; **bold:** human subunits; ***bold italics:*** mouse subunits. The Hill coefficient, GABA EC50, main conductance state and characteristic times of activation, deactivation and desensitization of the most abundant subtypes in vivo, i.e. ternary subtypes and ρ homopentamers, are presented. Information on other subtypes can be found in [13, 103, 112, 114, 116, 123, 125, 130, 146, 152–157, 176, 180, 182, 191, 192]

**Table 3:** Main electrophysiological parameters of the different GABA_A_ receptor subtypes in standard ionic conditions (subunits expressed in mouse L929 cells)

GABA_A_ receptor subtype	Single-channel conductance (pS)	GABA EC50 (µM)	Hill coefficient	Characteristic time of desensitization *t*_d_ (s)	Characteristic time of deactivation (ms)	Activation time (ms)
α1β1γ1		*5.2 *[165]				
α1β1γ2	30 [125] (γ2L splice variant) *29.3 *[145]* (γ2S splice variant)*	*7.1 *[146] *5.2 *[168]* (γ2L splice variant)* *5.1 *[169]* (γ2L splice variant)* 6.2 [125] (γ2L splice variant)	*1.7 *[146] *1.9 *[168]* (γ2L splice variant)* *1.9 *[169]* (γ2L splice variant)* 1.4 [125] (γ2L splice variant)		5 [125] (γ2L splice variant) *6.0 *[145]* (γ2S splice variant)*	
α1β1δ	22 [125]			No desensitization observed [125]	400 [125]	
α1β2γ2	*29 *[145]* (γ2S splice variant)*	11 [171]				
α1β3γ2	25.9 [114] 26.8 [130]	14 [171] 11.6 [130] (γ2L splice variant) 15.5 [111] (γ2L splice variant) 15.5 [182] (γ2L splice variant)	1.48 [111] (γ2L splice variant) 1.5 [182] (γ2L splice variant)	0.462 [114] ([GABA] = 1 mM, γ2L splice variant)	76.1 [114] (γ2L splice variant)	0.46 [114] (γ2L splice variant)
α1β3δ	23.8 [114] 26.7 [130]	2.8 [130] 4.4 [182]	1.4 [182]	1.26 [114] ([GABA] = 1 mM)	42.8 [114]	2.4 [114]
α1β3ε	24 [161] (rat α1 and β3, and human ε, in L929 cells)	0.8 [161] (rat α1 and β3, and human ε, in L929 cells)	0.9 [161] (rat α1 and β3, and human ε, in L929 cells)			
α2β3γ2		**25 **[111]** (γ2L splice variant)**				
α3β3γ2		35.8 [111] (γ2L splice variant)				
α4β3γ2		10.7 [111] (γ2L splice variant) **2.57 **[113]	**1.3 **[113]	**2.5 **[113]** ([GABA] = 100 µM)**		
α4β3δ		**0.5 **[113]	**1.3 **[113]	**4.8 **[113]** ([GABA] = 100 µM)**		
α5β1γ2		32.8 [157] (γ2L splice variant) 26 [124] (γ2L splice variant)	1.69 [157] (γ2L splice variant)			
α5β3γ2	22 [133] (γ2L splice variant)	5.7 [157] (γ2L splice variant) 6 [124] (γ2L splice variant) 9.4 [111] (γ2L splice variant)	1.54 [157] (γ2L splice variant)		51.8 [133] (γ2L splice variant)	0.9 [133] (γ2L splice variant)
α5β3γ3	26.9 [191]	1.5 [191]	1.6 [191]			
α5β3π	23.8 [191]	1.3 [191]	1.4 [191]			
α6β2γ2		2 [171]				
α6β2δ		0.2 [171]				
α6β3γ2		2 [171] (γ2L splice variant) 2.25 [111] (γ2L splice variant)	1.04 [111] (γ2L splice variant)			
α6β3δ		0.3 [171]				

Roman: rat subunits; **bold:** human subunits; *italics:* bovine subunits. The Hill coefficient, GABA EC50, main conductance state and characteristic times of activation, deactivation and desensitization of the most abundant subtypes in vivo, i.e. ternary subtypes and ρ homopentamers, are presented. Information on other subtypes can be found in [13, 103, 112, 114, 116, 123, 125, 130, 146, 152–157, 176, 180, 182, 191, 192]

**Table 4:** Main electrophysiological parameters of the different GABA_A_ receptor subtypes in standard ionic conditions (subunits expressed in *Xenopus* oocytes)

GABA_A_ receptor subtype	GABA EC50 (µM)	Hill coefficient
α1β1γ1	**25 **[163] *41 *[164] 75 [166]	
α1β1γ2	***9.8 ***[170]*** (γ2S splice variant)*** **19.9 **[126]** (γ2S splice variant)**	**1.36 **[126]** (γ2S splice variant)**
α1β1δ	***4.9 ***[170]	
α1β1ε	**4.0 **[126]	**0.85 **[126]
α1β2γ2	41 [153] 16 [132] (γ2L splice variant) 45.8 [47, 173, 174] 34 [97] (γ2L splice variant) 55 [175] **20 **[155] **20 **[163] ***4.61 ***[176]*** (γ2L splice variant)***	1.39 [153] 1.59 [174] 1.57 [47, 173] 1.38 [97] (γ2L splice variant) 1.4 [175] ***1.38 ***[176]*** (γ2L splice variant)***
α1β3γ2	**8 **[163]	
α2β1γ1	**39.8 **[183]	
α2β1γ2	**30.6 **[183]	
α3β1γ1	**114 **[163]	
α3β1γ2	240 [154] **208 **[163] **200 **[186]** (γ2S splice variant)** *98 *[164]	
α3β1γ3	**32 **[163]	
α3β1ε	**2.3 **[186]	
α3β1θ	**81 **[186]	
α3β2γ2	487 [154] **11 **[163]	
α3β3γ2	**28 **[163]	
α5β1γ2	17 [154] **15 **[163]	
α5β2γ2	14 [154]	
α5β3γ1	**24 **[163]	
α5β3γ2	**3 **[163]** (γ2L splice variant)**	
α5β3γ3	**2 **[163]	
α6β2γ2	6.7 [153] **1.6 **[155]	0.82 [153]
ρ1	**0.81 **[106]	**2.83 **[106]
ρ2	*1.19 *[162]	*2.17 *[162]

Roman: rat subunits; **bold:** human subunits; *italics:* bovine subunits; ***bold italics:*** mouse subunits. The Hill coefficient, GABA EC50, main conductance state and characteristic times of activation, deactivation and desensitization of the most abundant subtypes in vivo, i.e. ternary subtypes and ρ homopentamers, are presented. Information on other subtypes can be found in [13, 103, 112, 114, 116, 123, 125, 130, 146, 152–157, 176, 180, 182, 191, 192]

The electrophysiological properties of a ternary subtype are mainly determined by the α subunit isoform and the class of its “third subunit class” (Table 5, 6, 7). Few differences are observed between β or γ isoforms. Some of the differences observed between β isoforms are thought to stem from their differential distribution and association with different isoforms of the α class. For example, preferential association of β3 with α2 and α3 instead of α1 is likely to explain the slower kinetics of the β3-containing subtypes as compared with the other β-containing subtypes [193]. Moreover, the electrophysiology of a subtype does not result in a straightforward manner from the addition of the properties conferred by its subunits because of the complex interactions between these subunits: the properties of a subunit depend on its environment within the receptor.

**Table 5:** Properties conferred by the “first subunit class” of the receptor

isoform	α1	α2	α3	α4	α5	α6
GABA EC50	Medium	Medium	Very high	Medium	Medium	Very low
Desensitization	Complete and moderately fast	Complete and moderately fast	Slow and incomplete	Fast and complete	Slow and incomplete	Medium speed and complete
Refractory period	Long	Medium	Medium	Short	Short	Very long
Deactivation	Moderately fast	Moderately slow	Very slow	Very fast	Fast	Slow
Activation	Fast	Moderately fast	Slow	Medium	Moderately slow	Medium

**Table 6:** Properties conferred by the “second subunit class” of the receptor

Isoform	β1	β2	β3
GABA EC50	Medium	Medium	Moderate
Desensitization	Medium	Medium	Moderately slow
Deactivation		Medium	Moderately slow

**Table 7:** Properties conferred by the “third subunit class” of the receptor

Isoform	γ1	γ2L	γ3	δ	ε	π
Conductance	Medium	Medium	Medium	Low	Low	Medium
GABA EC50	Medium	Medium (γ2L variant) or moderate (γ2S variant)	Moderate	Low	Medium	Moderate
Hill coefficient	Medium	Medium	Medium	Medium	Low	Medium
Desensitization		Complete and moderately fast		Slow and incomplete	Fast	
Deactivation		Medium		Moderately fast		
Activation		Fast		Slow		

Binary αβ receptors display low GABA EC50, conductance and Hill coefficient, and activate slowly. In most cases, they desensitize slowly and not extensively, except the α1β3 subtype which desensitizes as fast and extensively as α1β3γ2L [114]. Despite substantial differences with ternary receptors, binary receptors helped to understand the molecular basis of ternary receptors electrophysiology. For example, using αβ2 subtypes, Olander et al*.* [115] showed that differences in activation and desensitization kinetics between α isoforms are determined by their transmembrane and intracellular domains. However, this study didn’t identify the cause of variations in deactivation kinetics, since the different αβ2 subtypes investigated didn’t differ significantly in that parameter. Similarly, the extent of desensitization of αβγ receptors was not matched by the corresponding αβ subtypes, with α1β2 desensitizing incompletely, contrary to α1β2γ. This confirms that novel properties emerge from the new subunits interactions introduced by γ or δ subunits in ternary receptors.

Receptors made of ρ subunits react very slowly to changes of GABA concentration, do not desensitize and deactivate quickly; they have low conductance and GABA EC50 but a high Hill coefficient.

The response of GABA_A_ receptors to particular GABA inputs can be predicted from the parameters that have been described so far. For example, due to similar conductance, faster activation and slower deactivation, α2β1γ2 mediates tenfold higher charge transfers than α1β1γ2 when it is activated [100], but the higher GABA EC50 of the former means that stronger inputs are necessary to activate it.

Several studies found a great consistency between the electrophysiological activity of in vitro recombinant and in vivo endogenous GABA_A_ receptors [60, 102, 103, 137, 152, 154, 194] (reviewed in [195]). The parameter values given above should therefore be relevant enough in physiological contexts to explain the activity and function of GABA_A_ receptors in the nervous system.

## GABA_A_ receptors mediate different types of currents at the neuronal scale

As receptors of the most abundant inhibitory neurotransmitter, GABA_A_ receptors are expressed in most neurons and influence their electric activity. Depending on the neuronal type, GABA_A_ receptors can be located in the dendrites, the cell body and/or the axons. They are highly enriched at post-synaptic sites [194, 196], where they usually mediate inhibitory neurotransmission, but can also be found extrasynaptically and perisynaptically. Despite the preferential synaptic localization of α1, α2, α3, and especially γ2 [197], no GABA_A_ receptor subunit has yet been found exclusively in synapses [196, 198]. Receptors containing the α4, α5, α6, γ1, γ3 or δ subunits, as well as binary αβ subtypes, are mainly extrasynaptic [56, 196, 197, 199], although the α4, α5 and α6 subunits can also be found in synapses at medium concentrations.

Due in part to the diversity in electrophysiology of the different subtypes, GABA_A_ receptors mediate several types of currents in neurons. These currents, and by extension the subtypes involved in their generation, have different biological functions.

### Phasic activity

Post-synaptic GABA_A_ receptors primarily have a phasic activity: they mediate intense currents of regulated duration upon binding GABA molecules released in the synaptic cleft by the pre-synaptic neuron. Phasic receptors are involved in synaptic communication and are the main mediators of inhibitory signals [200]. The most abundant synaptic receptors of the mature brain belong to subtypes containing the γ2 subunit associated with β subunits and α1 or α2 [114, 201]. Their electrophysiological properties (see Tables 5, 6, 7) ensure the speed and specificity of inhibitory post-synaptic currents (IPSCs) generated in response to pre-synaptic GABA release, ensuring efficient neuronal communication. Indeed, the GABA concentration reaches 2-5 mM in the synaptic cleft during a GABA burst [134, 202], a concentration much higher than the EC50 of any GABA_A_ receptor subtype (Tables 2, 3, 4). Low affinity receptors are thus activated by GABA bursts without being activated by background GABA concentration (Fig. 7a), which ensures that receptor activation is dependent on pre-synaptic inputs. The quick activation of most phasic receptors increases the speed at which neuronal networks communicate, and allows the receptors to respond to GABA bursts despite the extremely fast clearance of GABA in the synapse (whose time constant is only 0.3–0.6 ms) [134].

The transmission of a strong inhibitory signal requires maximization of charge transfer. This is ensured by the high receptor concentration (there are usually tens to hundreds of receptors per inhibitory synapse) [56, 73] and the maintenance of the IPSC for relatively long times [102]. Indeed, the slow or intermediate deactivation of phasic receptors enables IPSCs to last several tens of milliseconds. In addition, slow deactivation is associated with fast and complete desensitization, which explains the common observation of IPSC depression upon high frequency stimulation, a phenomenon termed rundown (Fig. 4a) [203, 204]. This process protects neurons against pathological excessive inhibition in case of defective clearance of GABA, or excessive GABA emission in the synaptic cleft.

Different GABA_A_ receptor subtypes can be located at different synapses of the same neuron. This phenomenon enables precise modulation of neurotransmission because identical signals received at different synapses will not induce the same phasic current in the postsynaptic neuron [196, 205, 206]. Several subtypes can even be expressed in the same synapse [207–210]. In such a case, the post-synaptic current triggered by GABA is a composite of the currents mediated by the individual subtypes.

Ternary subtypes (αβγ or αβδ) and ρ-containing subtypes are coexpressed at the axon terminals of retinal bipolar cells [208, 210] and mediate different types of phasic currents: ternary receptors mediate fast and brief currents, whereas ρ-containing receptors mediate long and delayed currents that can be explained by their slow activation and lack of desensitization [211]. Since ρ-containing subtypes have the lowest GABA EC50 (Tables 2, 3, 4), it is possible that at low GABA concentration, only the slow and long currents take place. Therefore, the diversity of phasic receptors facilitates complex and diverse responses to GABA inputs in a dose-dependent manner [5, 212]. In retinal bipolar cells, the contribution of different GABA_A_ receptor subtypes to the generation of inhibitory currents varies between cell subpopulations [213, 214], probably because of differences in abundance of the considered subtypes. Consequently, the IPSCs follow different times-courses in different cell subpopulations [215], leading to differences in cell function (reviewed in [5]). This exemplifies that the characteristics of an IPSC depend on the GABA_A_ receptor subtype by which it is mediated and that different types of neurons can vary in their phasic activity as a consequence of different subtypes expression patterns (reviewed in [216]).

During development, phasic currents are longer and less intense than in the adult brain [56, 73, 200, 217]. Indeed, the α1 and α2 subunits are not yet expressed at high levels and most synaptic receptors belong to the α3βγ2 subtypes, which display very slow deactivation (Tables 2, 3, 4).

**Fig. 6:**
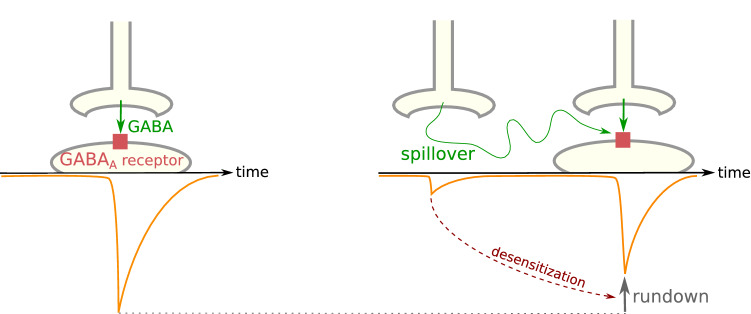
Effects of spillover on GABA_A_ receptor-mediated post-synaptic currents. Phasic activity occurs upon the fixation of GABA by GABA_A_ receptors on the post-synaptic neuron. Spillovers from nearby synapses can desensitize part of the receptors and decrease the amplitude of the next IPSC

Sometimes, GABA escapes from a synapse (a process termed spillover) and reaches a nearby synapse. The micromolar GABA concentration in the receiving synapse induces a very faint IPSC, and desensitizes a portion of the GABA_A_ receptors present there, which enter refractory periods that can last several seconds. If millimolar GABA bursts occur in the synapse before the end of the refractory period, the resulting phasic current will be diminished by rundown (Fig. 6) [131]. Since GABA bursts frequently occur in synapses, the net effect of spillovers is often excitatory despite the faint IPSC they directly induce. Spillovers are rare in physiological conditions in the adult brain since highly efficient GABA uptake systems usually ensure efficient clearance of GABA in synapses; however, these systems can be downregulated [218, 219].

Whether GABA_A_ receptor-mediated currents are excitatory or inhibitory depends in certain circumstances on receptor location and timing relative to depolarizing signals [220, 221], hence the importance of the brevity and spatial confinement of phasic currents [198]. Phasic currents are brief and often induced at a single synapse, allowing them to regulate neuronal and cerebral activity very finely and to generate a substantial diversity of responses to similar inputs, in part due to GABA_A_ receptor subtype diversity. Among other functions, GABA_A_ receptor phasic activity is required in the generation of θ and γ frequency network oscillations, under strict temporal control [198].

### Tonic activity

**Fig. 7:**
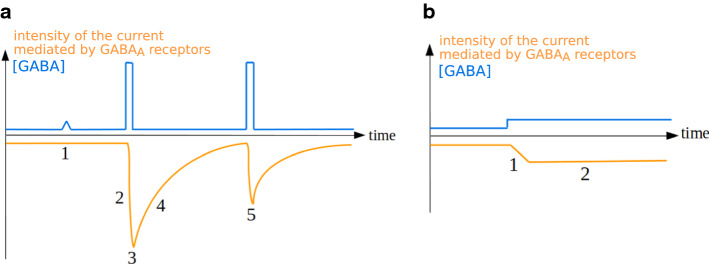
Comparison of phasic and tonic currents. **a** Phasic current. 1: due to the high GABA EC50 of most phasic receptors, background GABA transients do not activate GABA_A_ receptors. 2: fast activation. 3: high amplitude current peak. 4: slow deactivation. 5: rundown. **b** Tonic current. 1: slow activation. 2: steady-state, low-amplitude current with limited desensitization

Certain GABA_A_ receptors, mostly extrasynaptic, mediate chronic low-amplitude currents termed tonic currents [198]. Their activity is less temporally and spatially restricted than that of phasic receptors [198]. They are involved in long-run (seconds to minutes) modulation of neuronal activity and respond to GABA spillover or to non-synaptic release of GABA. They belong mostly to subtypes containing the δ subunit and the α4, α5 or α6 subunits [64, 114, 196, 222] or to ρ-containing subtypes [62], although certain γ2-containing subtypes can mediate tonic currents (reviewed in [201]). The characteristics of tonic activity are related to the electrochemical properties of the GABA_A_ receptor subtypes involved [223]: these subtypes display a high affinity to GABA (Tables 2, 3, 4), allowing a response to low extrasynaptic GABA concentration; and a low conductance as well as incomplete desensitization, enabling channel groups to deliver limited but steady currents (Fig. 7b). Furthermore, the amplitude of current mediated by δ-containing receptors at saturating GABA concentrations can be multiplied up to 20 times by the agonist etomidate [109], showing that the maximal open probability in the absence of agonists Pomax of the concerned subtypes is no higher than 5%, and explaining the low amplitude of the tonic currents they mediate.

γ-containing subtypes desensitize extensively when exposed to high GABA concentrations, but the low GABA concentrations which elicit tonic activity may not desensitize them to such an extent as to abolish tonic currents [114, 195]. Deactivation does not hamper tonic activity, because GABA presence is maintained over long periods, allowing closed receptors to reopen; deactivation even ensures that tonic currents remain at a low amplitude. Finally, the slow activation of tonic receptors, caused both by low GABA concentrations and the electrophysiological properties of the subtypes involved, participates in shaping tonic currents. At subsaturating GABA concentrations, the current peak is truncated, resulting in stable currents reaching a steady state without going through sharp peaks after changes in GABA concentration [112].

Receptors that usually mediate phasic currents can at times display a low-level tonic activity, including in synaptic locations, due to a very low but strictly positive probability of opening at GABA concentrations close to zero [132, 224]. For example, even at standard extracellular GABA concentration, the amplitude of tonic currents through α1β2γ2L can reach 1% of the amplitude of phasic currents [224]. Moreover, it has been proposed that certain subtypes can mediate both phasic and tonic currents not only depending on their location or on GABA concentration, but also on whether both or only one of their two GABA-binding sites are occupied [175].

Tonic charge transfer can have a significant effect on neuronal communication: it has stronger inhibitory effects than phasic inhibition in certain neuron types such as cerebellar granule cells and hippocampal pyramidal cells [73, 200, 222, 225]. This demonstrates that the steadiness of tonic currents can compensate for their lack of intensity compared to phasic currents in generating a strong response [222].

Tonic activity has been predicted to have different effects than phasic activity on neuronal network activity, notably a decrease of rhythmicity and synchronicity between neurons [226, 227]. At a wider scale, models of cerebellar activity predict that tonic inhibition increases the number of motor patterns that can be stored in the cerebellum [228, 229].

During development, tonic activity is mainly activated by action potential-induced GABA release in the extracellular space [73, 200, 217]; however, non-vesicular and acetylcholine-induced, action potential-independent GABA release plays the major role in tonic inhibition in the adult cerebellum [137, 200]. This developmental transition can be related to the increasing wrapping of synapses by glial cells [230], which progressively prevents GABA spillover [200]. It is not only the source of tonic currents that varies during development, but also their amplitude. For example, tonic currents are detected in hippocampal pyramidal cells only in embryonic life [231], while they increase over time in cerebellar granule cells [73, 200]. Spatial discrepancies can also be caused by variations in extracellular GABA concentration between brain areas [225].

Tonic currents can modify the information conveyed by neurons [70, 232]. Unlike phasic currents, they do not determine the information conveyed by a single message but rather subtly modulate the information transmitted by the neuron over long time frames encompassing several communication events. Tonic inhibition is a form of regional-scale neuromodulation, offering a negative feedback to periods of intense synaptic activity during which successive spillovers increase the extracellular GABA concentration [198]. The long duration of tonic currents enables them to mitigate the oscillatory patterns often induced by phasic communication [226] and thus to desynchronize the activity of neighbor neurons [227].

Since the various tonic GABA_A_ receptor subtypes differ in their EC50, slight increases in GABA extracellular concentration can progressively recruit a new receptor population. It widens the repertoire of potential tonic activity, since limited and finely regulated changes in GABA concentration can lead to the selective activation or inhibition of only part of the receptors mediating tonic currents [225].

Most transporters responsible for the clearance of GABA out of the extracellular space are unable to lower GABA concentration below 0.4 µM [233], a concentration higher than the EC50 of several α6-containing subtypes (Tables 2, 3, 4). Some receptors are thus continually activated and exert a function of leakage channel, as experimentally confirmed in the rat hippocampus [234]. Rather than being involved in the transmission of inhibitory signals, such receptors are believed to regulate membrane resistivity [78]. If this hypothesis is correct, these receptors control the time window over which synaptic integration occurs as well as the neuronal gain (the ratio between received and emitted currents), and thus the action potential firing rate [225, 232]. This exemplifies that the functions accomplished by a GABA_A_ receptor subtype are determined by its electrophysiology.

### Perisynaptic GABA_A_ receptors

Perisynaptic GABA_A_ receptors are activated by GABA spillover out of the synaptic cleft. They account for the slow-component inhibition of biphasic GABA-mediated response [235], a phenomenon in which the GABA released in a synapse first activates phasic post-synaptic receptors, then spills over in the extracellular space and activates perisynaptic receptors. GABA diffuses in a much greater volume in the extracellular space than in the synaptic cleft. Consequently, it reaches perisynaptic receptors with a delay compared to synaptic receptors and at a much lower concentration. Therefore, the biphasic GABA-mediated response consists of a localized, fast and intense phasic current followed by a diffuse, low-level and less cell-specific hyperpolarization. Perisynaptic receptors thus prolong phasic activity. They also take part in tonic inhibition [73] and spillover-mediated heterosynaptic modulation [232], whereby GABA spillover out of a synapse inhibits nearby neurons uninvolved in the considered synapse (reviewed in [236]).

### Presynaptic inhibition

GABA_A_ receptors can be found on the axons of certain neuron types, where they sometimes mediate depolarizations, with either inhibitory [237, 238] or excitatory [78, 239] effects on downstream synaptic activity.

**Fig. 8:**
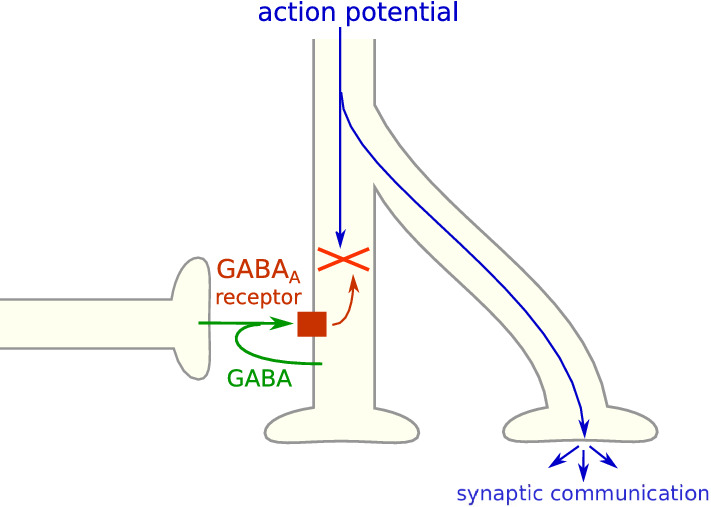
Presynaptic inhibition of action potentials mediated by GABA_A_ receptors. Inhibitory currents mediated by GABA_A_ receptors can inhibit simultaneous action potentials, and allow different axonal ramifications to convey different signals

In the process of presynaptic inhibition, GABA released at axo-axonic synapses activates GABA_A_ receptors on the axon of the receiving neuron and prevents the propagation of synchronous action potentials [240] (Fig. 8). Unlike synaptic and perisynaptic inhibitions [241], presynaptic inhibition requires a precise synchronization of the excitatory signal with the chloride influx mediated by the GABA_A_ receptor [198]. However, this does not necessarily imply that the GABA input must be synchronized with the action potentials: indeed, in certain retinal cells where ρ-containing subtypes are exclusively located in axons [213], the long-lasting signals mediated by these subtypes ensure an efficient presynaptic inhibition which allows a small delay between the inhibitory input and the action potentials.

The functions of presynaptic inhibition remain poorly understood [78]. However, the release of GABA by axons of hippocampal mossy fiber [242, 243] or cerebellar basket and stellar cells [244] suggests that one function of presynaptic inhibition is self-inhibition and tissular negative feedback on neuronal activity. In addition, presynaptic inhibition enables the axonal ramifications of a neuron to convey different messages if only part of the ramifications receive a presynaptic inhibitory signal (Fig. 8). This mechanism increases the complexity and specificity of neuronal communication [245]. For example, the presynaptic inhibition mediated by ρ-containing subtypes in the retina increases the dynamic range of brain responses to light intensity [246].

## Control of GABA_A_ receptor electrophysiology

GABA_A_ receptor electrical activity is determined by GABA concentration and its intrinsic electrophysiological properties, but also by its chemical and cellular environment, sometimes in a subtype-specific manner.

### GABA_A_ receptor control by phosphorylation

GABA_A_ receptors are subject to phosphorylation by several kinases such as protein kinase A (PKA) and C (PKC). Phosphorylation by PKC inhibits GABA_A_ receptors, probably by endocytosis [247] or through an inhibition of the non-desensitizing fraction of GABA_A_ receptors [248]. Both increases [249, 250] and decreases [147, 251–253] in currents mediated by GABA_A_ receptors have been reported upon phosphorylation by PKA, probably because it can simultaneously speed up desensitization while slowing down deactivation [254, 255]. In the retina, PKA mediates dopaminergic signals which result in a potentiation of GABA_A_ receptors through a decrease in GABA EC50 [128]. Phosphorylation may explain the differences in electrophysiology between γ2S and γ2L containing receptors, since the only difference between these two splice forms is a cytoplasmic insertion of 8 residues in γ2L containing a PKC phosphorylation site [34].

Receptors of different neurotransmitters can colocalize at post-synaptic sites and be activated by presynaptic co-release of several neurotransmitters. The simultaneous activation of different postsynaptic receptors often induces a partial occlusion of their respective currents. This cross-talk has been proposed to represent a fast adaptive process in controlling signal transmission [256]. Negative cross-talk was demonstrated between GABA_A_ receptors and dopamine receptors [256], P2X receptors [257] and glycine receptors [258]. The inhibition of GABA_A_ receptors by glycine receptors depends on phosphatase 2B activity, while kinase activity is necessary for recovery of GABA_A_ receptors from this inhibition [259].

Glutamate excitatory neurotransmission mediated by NMDA receptors induces calcineurin-dependent γ2 subunit dephosphorylation, leading to a reversible and local dispersion of postsynaptic GABA_A_ receptor clusters [260]. Dispersion lowers receptors’ EC50, activation and desensitization times, and slows down their deactivation [261–263]. Consequently, receptors transition from synaptic phasic activity to extrasynaptic tonic activity upon dephosphorylation.

### Voltage dependency

Although GABA_A_ receptors are generally voltage-independent, a few subtypes are voltage-sensitive in rare situations [123, 240, 264]. Voltage seems to affect current peak amplitude but not kinetic properties (desensitization and deactivation) of the α1β3δ, α5β3π and ρ1 subtypes [106, 123, 191, 192], unlike the α6β3δ subtype whose kinetic properties are affected but not other parameters [123]. Voltage dependency can also be observed in channels that are rare or never found in vivo, such as α1β3 or α6β3 [123].

### Ionic modulation

**Fig. 9:**
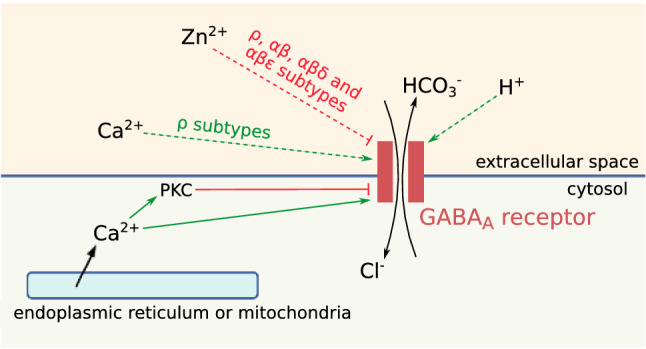
Ionic modulation of the GABA_A_ receptor. Ion flows are represented with black arrows, activation with green arrows and inhibition with red arrows. Dashed lines indicate subtype specificity

GABA_A_ receptors are sensitive to ionic modulation: a change in the concentration of certain ions, in either the extracellular or the cytosolic compartment, can modify their activity. The nature or extent of the modulation is subtype-specific (Fig. 9).

Extracellular Ca^2+^ does not affect the activity of ternary receptors [87, 265], but it increases the amplitude of currents mediated by ρ-containing subtypes [266]. Intracellular Ca^2+^ has varying effects on many subtypes, depending on its origin and concentration. Ca^2+^ coming from the extracellular space does not affect GABA_A_ receptor opening [267], whereas Ca^2+^ originating from intracellular compartments activates GABA_A_ receptors at low concentrations, but inhibits it at high concentrations [268, 269]. This may be a consequence of receptor phosphorylation by PKC [248] or dephosphorylation by calcineurin [260], because these enzymes are activated by intracellular calcium. Ca^2+^ control of GABA_A_ receptors is involved in the long-run (seconds to minutes) modulation of neuronal activity [268].

Extracellular Zn^2+^ is released during synaptic activity by numerous neurons, particularly in the limbic and neocortical regions [270] and during development. It decreases the opening frequency of certain GABA_A_ receptor subtypes [271] (reviewed in [4]). αβ receptors are up to 3400-fold more sensitive to Zn^2+^ than αβγ and αβδ receptors [113, 272], while ρ [266], αβπ [191] and αβε [126, 171] subtypes have an intermediate sensitivity. In addition, α1 confers a higher zinc sensitivity than the other α subunits [124, 126, 171, 182, 189, 273]. The discrepancy in sensitivity between αβγ and αβ subtypes is explained by the position of the Zn^2+^ binding sites in the receptor (two between α and β subunits and one in the ion pore), which are disturbed by γ subunits [182, 274]. Additionally, αβ subtypes comprised of two α and three β subunits contain a third zinc-binding site at the β-β interface, explaining their higher sensitivity compared with αβγ subtypes and the very rare αβ subtypes containing three α and two β subunits [129]. Zinc increases the EC50 of α1β1δ but not α1β1, and the Hill coefficient of α1β1 while it decreases the Hill coefficient of α1β1δ [273]. Zn^2+^ sensitivity displays very similar characteristics in vivo and in vitro (reviewed in [5]). Zn^2+^ may take part in the control of tonic currents in hippocampal pyramidal neurons, where up to 10% of extrasynaptic GABA_A_ receptors may belong to αβ subtypes [180].

All GABA_A_ receptor subtypes seem to be insensitive to intracellular pH [275] (reviewed in [4]), but certain subtypes are sensitive to extracellular pH because of their two extracellular proton-binding sites [170, 275]. Conductance is negatively correlated with extracellular pH for α1β1, α1β2γ2 and α1β1δ [170, 276], and positively for ρ subtypes [277, 278], while α1β1γ2S is pH-insensitive [170] and the curves of α1β1γ2Sδ, α4β3γ2, α4β3δ and α1β2γ2S conductance as a function of extracellular pH are bell-shaped [73, 170]. Extracellular protons slow the deactivation of the α1β3δ subtype and increase its opening probability in the absence of GABA without affecting its desensitization, resulting in an increase of currents at low pH [181]. On the contrary, α1β2γ2 desensitizes less and slower at low pH [276], while its activation is slowed, and deactivation is shifted toward its slow component. GABA_A_ receptor inhibition at high pH is explained by a faster closure of the channel [279].

In vivo, GABA_A_ channel charge transfer is generally negatively correlated with extracellular pH [170, 280]. During synaptic transmission, a transient acidosis of the synaptic cleft is followed by a durable increase in pH [281]. This phenomenon synergizes with the concomitant variations of GABA concentration and helps to induce a fast and massive opening of GABA_A_ receptors during the first milliseconds of synaptic transmission, followed by the progressive closure of the channels when protons and GABA are cleared from the synaptic cleft.

NMDA, the glutamate receptor mediating most of the excitatory signals in the central nervous system, is inhibited by low pH [282]. Thus, the opposite effects of pH on the inhibitory GABA_A_ receptor and the excitatory NMDA receptor may explain why neuronal activity and excitability increase with extracellular pH [283]. High regional electrical activity elicits an acidosis: the pH-sensitivity of GABA_A_ receptor and NMDA receptor thus creates a negative feedback against hyperactivity. This process relies notably on tonic currents mediated by δ-containing receptors [181]. Other pH regulation processes can also modulate neurone activity through GABA_A_ receptors [280]. Extracellular pH can itself be modified by bicarbonate transport across GABA_A_ receptors [284], making them broadcasters of neuromodulatory signals in addition to their function of chloride channel.

Furthermore, acidity displays protective effects against Zn^2+^ inhibition in α1β1 receptors [273], showing that the different types of ionic modulation to which GABA_A_ receptors are submitted are not independent.

The ionic modulation of the GABA_A_ receptor is involved in the long-term depression or potentiation of inhibitory currents characteristic of neuronal plasticity, and may play a role in hypomagnesia-induced hyperexcitability [285], anoxic and ischaemic conditions [286] or epileptogenesis [287].

## Conclusion

GABA_A_ receptors are highly complex and are able to generate a vast repertoire of electric responses to different inputs under the control of various types of regulations. Their influence on cellular electric activity at different scales of time and space can to a great extent be predicted from the structural and electrophysiological properties of the different subtypes, whose diversity provides additional modularity, robustness and specialization to the GABA_A_ receptor system.

GABA_A_ receptor agonists and antagonists are increasingly used in medicine to treat various diseases, from epilepsy to insomnia and others. Improved knowledge of the electrophysiology, pharmacology and expression patterns of GABA_A_ receptor subtypes has led to the development of subtype-specific drugs that limit side effects. Further studies on GABA_A_ receptor electrophysiology are warranted in order to develop new treatments, particularly in the fields of GABA_A_ receptors structure–function relationships, interactions with intracellular proteins, and electrical properties of lesser-known subtypes such as those containing π or θ subunits.

## Data Availability

All data relevant to this review is included in the text, references and figs.
